# Symbiotic microbial studies in diverse populations of *Aphis gossypii*, existing on altered host plants in different localities during different times

**DOI:** 10.1002/ece3.8100

**Published:** 2021-09-23

**Authors:** Shuai Zhang, Honghua Su, Weili Jiang, Daowu Hu, Intazar Ali, Tianxing Jin, Yizhong Yang, Xiaoyan Ma

**Affiliations:** ^1^ School of Horticulture and Plant Protection Yangzhou University Yangzhou China; ^2^ Basic Experimental Teaching Center of Life Sciences Yangzhou University Yangzhou China; ^3^ State Key Laboratory of Cotton Biology Institute of Cotton Research Chinese Academy of Agricultural Sciences Anyang China; ^4^ Department of Entomology, Faculty of Agriculture and Environment (FA & E) The Islamia University of Bahawalpur, Baghdad ul‑jadeed Campus Bahawalpur Pakistan

**Keywords:** abundance, cotton aphid, geographical distribution, infection frequencies, symbiotic bacteria

## Abstract

Complex interactions between symbiotic bacteria and insects ultimately result in equilibrium in all aspects of life in natural insect populations. In this study, abundance of principal symbiotic bacteria was estimated using qPCR in 1553 individuals of aphids, *Aphis gossypii*. Aphids were sampled from primary and secondary host plants—hibiscus and cotton. Hibiscus aphids were collected from 24 different locations in April, September, and November, whereas cotton aphids were collected between 2015 and 2017 from areas with wide variations in climatic conditions. About 30%–45% aphids were recorded with the most dominant symbiont, *Arsenophonus*. The other symbionts were in low frequency, and about 7% of aphids were noted with *Hamiltonella*, *Acinetobacter*, and *Microbacterium*, and 3% of aphids were verified with *Serratia* and *Pseudomonas*. Aphids infected with *Hamiltonella*, *Arsenophonus*, and *Serratia* can influence *Buchnera* densities. *Hamiltonella* has positive interaction with densities of *Arsenophonus* and *Serratia*. Almost 100% coinfection of *Hamiltonella* and *Arsenophonus* was detected in Xinxiang aphids and 50% coinfection was reported in aphids from North China, while no coinfection was detected in Hainan aphids. These findings describe the prevalence pattern and richness of core community of symbiotic bacteria in naturally occurring populations of *A*. *gossypii* and provide new insights for the study of symbiotic bacteria.

## Graphical abstract

About 30%–45% and 10% aphids were found infected with *Arsenophonus* and *Hamiltonella*, respectively. Aphids infected with *Hamiltonella*, *Arsenophonus*, and *Serratia* can influence *Buchnera* densities with different variations. Host plant may play important role than biotypes on bacterial symbiont infection frequencies and densities.

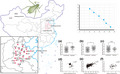

## 1 INTRODUCTION

**FIGURE 1:**
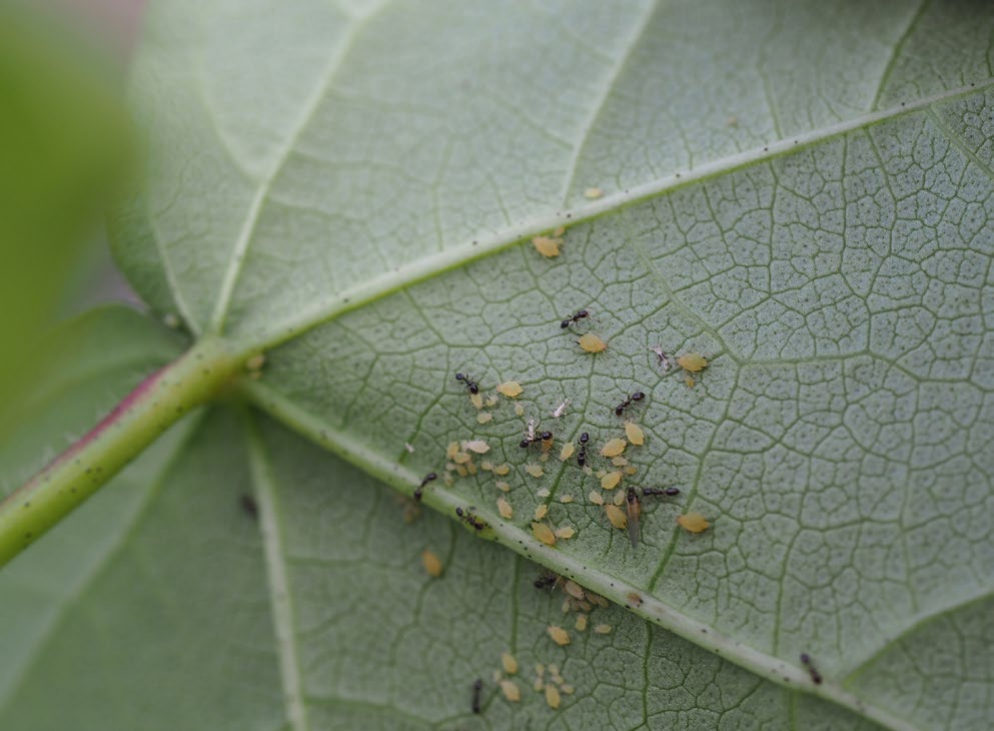
The mutualistic interaction between *Aphis gossypii* and ants on cotton. The cotton aphid, *A. gossypii* Glover, is an important global pest that sucks sap and transmits viral diseases to host plants, causing serious economic losses in agriculture. Due to the difference in the life cycle and other factors, such as the mutualistic interaction between cotton aphids and ants, the synergy between symbiotic bacteria and aphids is quite different within aphid species or different in populations of same species. In this study, seven bacterial abundances in 1553 *A*. *gossypii* were estimated using quantitative PCR, providing novel information about bacterial community related to biotypes, host plants, prevalence time, and geography

The cotton aphid, *Aphis gossypii* Glover, is an important global pest that sucks sap and transmits viral diseases to host plants, causing serious economic losses in agriculture (Figure 1) (Blackman & Eastop, 2000). It colonizes more than 600 plant species, many of which are important crops (Ebert & Cartwright, 1997). Plant transfer experiments and genetic diversity analysis have highlighted the existence of *A*. *gossypii* host biotypes (Carletto et al., 2009; Wang et al., 2016; Zhang et al., 2018). *A*. *gossypii* is distributed on a large geographical scale with a wide range of host plants, which provides a basis for a highly variable life cycle, with a holocyclic pattern in cold winters and anholocyclic forms in warm regions (Margaritopoulos et al., 2006).

*Aphis gossypii* usually migrates between the primary and the secondary hosts throughout the year (Kwon & Kim, 2017; Xia et al., 1999). In many areas, *A*. *gossypii* uses hibiscus plants as the primary host, between April and mid‐May; then migrate to secondary host plants; and again return to feed on hibiscus from October to November, whereas some aphids eat hibiscus all the year long (Charaabi et al., 2008; Zhang & Zhong, 1990).

Symbiotic bacteria exist in many insects, known as primary symbionts (obligate symbionts), secondary symbionts, and facultative symbionts (Bright & Bulgheresi, 2010). Symbiotic bacteria have conditionally positive effects on the physical condition of the host insects. During host adaptation, bacterial symbionts suppress plant resistance (Su et al., 2015) or increases the expression of detoxifying enzymes (Singh et al., 2020) that efficiently enhance the host adaptation. Recent studies showed that symbiotic bacteria contribute to host variation, heat tolerance (Zhang, Leonard, et al., 2019), and temperature preference (Hague et al., 2020). Meanwhile the host should also bear the metabolic and fitness‐dependent cost to symbiont bacterial presence (Engl et al., 2020; Oliver et al., 2008); bacterial symbionts even can shape their host evolution (Coffman & Burke, 2020). Therefore, it is very important and necessary for insects to maintain a balanced population of symbiotic bacteria.

In primary symbionts, *Buchnera* is essential for sap‐sucking aphids—reported in almost all aphid species, and responsible for essential amino acids and other nutrients for growth, reproduction (van Ham et al., 2003), and to improve heat tolerance (Zhang, Leonard, et al., 2019). The interaction of many facultative symbionts within aphids were well‐studied, such as the bacterium *Hamiltonella* increases resistance to its parasitoid via toxin protein (Brandt et al., 2017) and *Rickettsiella* changes body color from red to green in its natural populations (Tsuchida et al., 2010).

Due to the difference in the life cycle, host type, pesticide selection pressure, dispersal, and migration, the synergy between symbiotic bacteria and aphids is quite different within aphid species or different in populations of same species. Some studies have been carried out in bacterial communities of *A*. *gossypii* based on 16S rRNA gene sequencing on Illumina platforms (Gallo‐Franco et al., 2019; Xu et al., 2020; Zhang, Luo, Wang, et al., 2019; Zhao et al., 2016), quantitative PCR, and normalized host genes (Ayoubi et al., 2020; Chong & Moran, 2016; Zhang, Cao, et al., 2016). These findings reflect that symbiotic bacteria play an important role in *A*. *gossypii*, but the information about absolute quantity of symbiotic bacteria in natural populations and their changing trends within seasons, geographic areas, and hosts is still lacking. In this study, seven bacterial abundance in 1553 *A*. *gossypii* were estimated using quantitative PCR (qPCR), providing novel information about bacterial community related to biotypes, host plants, prevalence time, and geography.

## 2 MATERIAL AND METHODS

### 2.1 Field sampling and DNA extracting

For Illumina MiSeq DNA sequence analysis, wingless *A*. *gossypii* were collected from cotton fields located in Anyang (Henan Province) and Shihezi (Xinjiang Province) in late June 2016. Different instars of wingless aphids were picked up from cotton and then put in nuclease‐free Eppendorf tubes. In order to make the sample data representative, only one aphid per cotton plant in each sampling field was collected. In this way, more than 50 aphids from 50 different fields were collected, mixed, and considered as one sample; 10 samples from each city were collected and immediately immersed in liquid nitrogen and stored at −80°C for further study.

For qPCR analysis, *A*. *gossypii* were collected from cotton and hibiscus plants, each adult wingless aphid was put in a nuclease‐free Eppendorf tube. (a) From cotton, *A*. *gossypii* were collected from Xinjiang (2015) and Henan (2015–2017) in June and from Hainan (2015) Province in February. Only one wingless adult aphid per plant from 8 to 48 cotton plants per site was collected to avoid sampling from the offspring of a single female (Zhang et al., 2018); aphids were immersed in liquid nitrogen and stored at −80°C for further study. (b) From hibiscus, *A*. *gossypii* were collected from ornamental hibiscus located in urban areas from each location; the locations are shown in Appendix S1. Aphids were collected in April, September, and November 2016. In order to avoid the confounding of the area caused by the transplanting of seedlings, we choose hibiscus which has been transplanted more than two years. Only one aphid per plant was sampled, and the next sample was collected from plants more than 10 meters away from the previous plants from where the sample was drawn. Sampled aphids were placed in 95% ethanol and stored in room temperature for further study.

Each sample was washed with 70% ethanol and rinsed three times with nuclease‐free water, and total DNA from individual aphids or mixed samples was extracted using the TIANamp Genomic DNA Kit (TIANGEN Biotech (Beijing) LTD., China) according to the manufacturer's instruction. In order to break gram‐positive bacterial cells, additional lysozyme (50 mg/ml) was added at the incubation step. Elution buffer, 30 μl, was added at the last step, and re‐elution was done for one more time. Negative DNA extraction (control) includes DNA extractions of the nuclease‐free water. The quantity and quality of the DNA were measured with a NanoDrop 2000C spectrophotometer (Thermo Scientific). The purified DNA samples were stored at −20°C, and the samples having lower concentration, <25 ng/μl, were excluded from the study.

### 2.2 16S rRNA gene amplification and sequencing

The V3‐V4 hypervariable regions of the bacterial 16S rRNA gene were amplified using the primers 338F (5′‐ACTCCTACGGGAGGCAGCAG ‐3′) and 806R (5′‐GGACTACHVGGGTWTCTAAT‐3′). DNA from mixed aphid samples was used as template DNA. Amplicon generation of PCR products, quantification and qualification, PCR product mixing and purification, library preparation, and sequencing were carried out on an Illumina MiSeq platform at Shanghai Major Bio‐pharm Technology Co., Ltd. Bioinformatics. Sequences with ≥97% similarity were assigned to the same OTUs.

### 2.3 Quantification of symbiotic bacteria

qPCR was used to determine copies of 16S rRNA genes of dominant bacteria, and entire DNA from individual aphids—diluted 10 times—was used as template DNA. Bacterial special primers (Appendix S2) were designed according 16S rRNA genes using BEACON DESIGNER 7.6 (PREMIER Biosoft International, CA, USA). Primer PCR efficiencies were tested using series‐diluted templates (Appendix S2). The standard template was prepared, as previously reported (Zhang, Luo, Jiang, et al., 2019), and the general step was cloning the target sequence in a plasmid of pEASY‐T3 cloning vector (TransGen Biotech, China). *Escherichia coli* DH5α was used as a host for plasmid propagation, and the target sequence in the plasmid vectors was confirmed by sequencing. Each PCR reaction contained 5 μl 2×TransStart Green qPCR SuperMix (TransGen Biotech, China), 0.2 μl each of 10 mM forward and reverse primers, 2.0 μl template DNA (2.0 μl negative DNA considered as negative controls), and 0.2 μl 50 × ROX; nuclease‐free water was added to make up to 10 μl. The Step OnePlus™ Real‐Time PCR System (Applied Biosystems, Foster City, CA, USA) was used to perform PCR, according to cycling conditions of 95°C for 3 min followed by 40 cycles of a two‐step PCR (95°C for 5 s, 60°C for 30 s). All qPCR reactions were done in triplicate for each individual aphid DNA, and each reaction plate generated a corresponding standard curve. To make sure the primer specificity validated for each primer, checking steps were carried out, as previously reported (Zhang et al., 2019).

### 2.4 Statistical analysis

Bioinformatics of 250‐bp paired‐end reads were conducted, as previously reported (Zhang, Luo, Jiang, et al., 2019). Principal component analysis (PCA) based on the Bray–Curtis method was performed to study alpha diversity (ACE and Chao1 estimators, Good's coverage estimates, and Shannon and Simpson diversity indices) among observed species. Alpha diversity and beta diversity analyses were executed based on normalized output data by random selected sequences per sample according to the sequence number of the sample with the least sequences, and the statistical analyses were carried out by using the independent two‐sample *t* test to identify the differences between two groups, at *p* < .05, which is considered significant. All analyses and estimates were carried out on the freely accessible online Majorbio Cloud Platform (https://cloud.majorbio.com).

The abundance of symbiotic bacteria in one microliter of DNA solution was estimated by measuring the 16S rRNA gene of each bacterium. Standard curves were generated using series dilutions of the standard plasmid vector, and 6 proportional dilutions of the plasmid were prepared with the lowest concentration, composed of nearly 100 copies. Due to addition of lowest concentration, the standard curve cannot be constructed well. Other 5 dilutions of standard plasmid vectors were used as a result of aphid's abundance lower than the lowest point of the standard curve and considered as undetectable bacterial individuals.

The analysis of similarity (ANOSIM) and permutational multivariate analysis of variance (PERMANOVA) revealed that the most important biological factor (hosts, seasons, and geography) effected on symbiont and secondary symbiont community structures. ANOSIM and PERMANOVA were performed using Adonis and Anosim functions in R vegan package (version 2.5‐7, https://cran.r‐project.org/web/packages/vegan/), respectively, based on the Bray–Curtis community dissimilarity index with 999 permutations. SPSS 20.0 was used to evaluate the differences between infection frequency and abundance. For comparison of infection frequency between two samples, Pearson's chi‐squared test was used when *n* ≥ 40, and Fisher's exact test was used when *n* < 40. Group comparisons of symbiotic bacteria and abundance were evaluated with the Mann–Whitney U test (group number > 2) and Kruskal–Wallis test (*n* > 2). Correlation between different bacteria was carried out using the Pearson correlation of the means of these parameters.

## 3 RESULT

### 3.1 Overview of the bacterial diversity from Henan and Xinjiang

The V3‐V4 region of 16S rRNA gene was amplified from *A*. *gossypii* samples collected from Henan and Xinjiang provinces, and the Illumina MiSeq PE300 platform was used to generate raw reads. There were 78206–140328 raw reads from Henan samples, and 86988–145634 raw reads from Xinjiang‐collected samples. Good's coverage estimates of sequencing data were noted with maximum coverage in all samples, remained more than 99% (Appendix S3). The quality filtering sequences for aphids from Henan and Xinjiang was assigned 274 and 453 OTUs containing 170 co‐existed OTUs, respectively.

**FIGURE 2:**
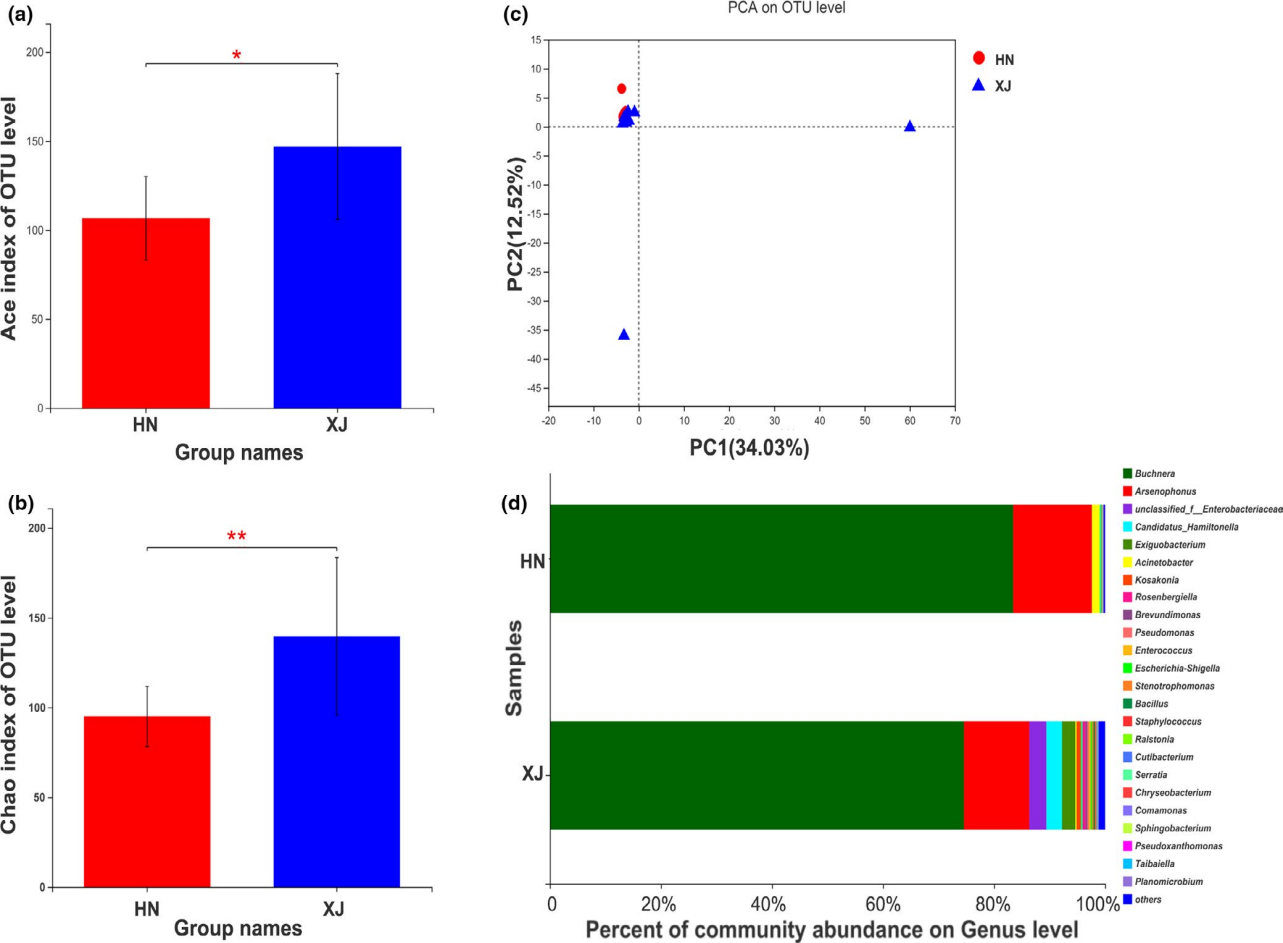
Bacterial diversity of *Aphis gossypii* from Henan (HN) and Xinjiang (XJ) provinces by high‐throughput sequencing approaches. Community richness and diversity measured by Ace (a) and Chao1 (b), Student’s *t* test was used for analysis significant differences of group mean value, *p < .05 and **p < .01. Principal component analysis based on the 20 samples (c) and symbiotic bacteria relative abundance of the bacteria in *A. gossypii* on genus level (d)

Xinjiang‐collected aphids were found with higher bacterial biodiversity estimates (ACE and Chao1) than the aphids collected from Henan. Aphids from Xinjiang were also found with higher diversity of bacterial species (Figure 2a,b). The PCA‐based studies of the 20 samples showed all clusters together, except two Xinjiang samples (Figure 2c). The most abundant symbiotic bacterial phylum was Proteobacteria, which accounted for 99.76% and 96.04% in Henan and Xinjiang samples, respectively. The most abundant genus was the primary symbiont *Buchnera*, having a relative abundance of 83.78% in aphids from Henan and 74.93% in aphids from Xinjiang. Generally, *Arsenophonus*, *Acinetobacter*, *Serratia*, *Brevundimonas*, and *Pseudoxanthomonas* were the top 5 most abundant facultative symbionts at the genus level in aphids from Henan, which accounted for 15.65% of the total. The bacterial genera *Arsenophonus*, *Hamiltonella*, *Exiguobacterium*, and *Kosakonia* including unclassified genus of the family *Enterobacteriaceae* were the 5 most abundant facultative symbionts in aphids from Xinjiang and accounted for 20.87% of the total (Figure 2d).

### 3.2 The relationship between symbiotic bacteria

The infection frequency and abundance of 7 main symbiotic bacteria in single *A*. *gossypii* were analyzed by qPCR. From 1533 *A*. *gossypii* collected from North China—Xinjiang and Hainan provinces—100% individuals were found with primary symbiont *Buchnera*, 31.51% individuals were noted by at least one facultative symbiont, and 11.87% individuals were observed with two or more symbionts. *Arsenophonus* was the most prevalent facultative symbiont, and 29.94% individuals were perceived with it, followed by *Acinetobacter* (7.31%), *Hamiltonella* (7.11%), *Microbacterium* (6.91%), *Serratia* (3.26%), and *Pseudomonas* (2.22%). During this study, it was noticed that 56.62% individuals were not found by any facultative symbiont.

**FIGURE 3:**
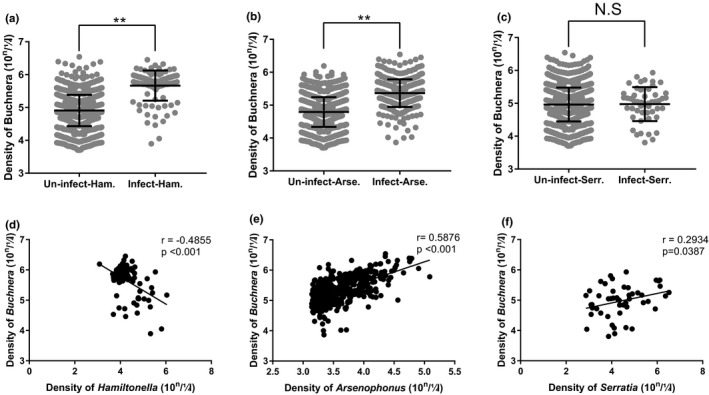
The density of *Buchnera* and its relationship with facultative symbionts. The *Buchnera* copy numbers in one microliter of aphid DNA were show in a. (*Hamiltonella*‐infected aphids (Infect‐Ham.) vs. uninfected aphids (Un‐infect‐Ham.)), b. (*Arsenophonus*‐infected individuals (Infect‐Arse.) vs. uninfected individuals (Un‐infect‐Arse.)), c. (*Serratia*‐infected individuals (Infect‐Serr.) vs. uninfected individuals (Uninfect‐Serr.)), scatter dot plot with mean ± standard deviation. **p < .01 and N.S = no significant difference, data were analysis by Mann–Whitney *U* test. The correlation between copy numbers of *Buchnera* and *Hamiltonella* (d), *Arsenophonus* (e), and *Serratia* (f) were test according to bacteria copy numbers in one microliter of aphid DNA

The density of *Buchnera* was influenced by some facultative symbionts. Both *Hamiltonella‐* and *Arsenophonus*‐infected aphids have higher density of *Buchnera* than the uninfected ones (Mann–Whitney‐*U* tests, *p* < .001) (Figure 3a,b). In *Hamiltonella*‐infected aphids, *Buchnera* density was negatively correlated with the *Hamiltonella* density (*r* = −.4855, *p* < .001) (Figure 3d), and *Buchnera* density was positively correlated with *Arsenophonus* density in *Arsenophonus* infected aphids (*r* = .5876, *p* < .001) (Figure 3e). Interestingly, *Buchnera* density has no significant difference between *Serratia*‐infected aphids and *Serratia*‐uninfected aphids (Mann–Whitney‐*U* tests, *p* = .611) (Figure 3c), but when aphids were infected with *Serratia*, *Buchnera* density was clearly correlated with *Serratia* density (*r* = .2934, *p* = .0387) (Figure 3f). *Buchnera* density was not remained significant, between *Acinetobacter*‐infected aphid and ‐uninfected aphids (Mann–Whitney‐*U* tests, *p* = .890), among *Microbacterium*‐infected aphids and uninfected aphids (Mann–Whitney‐*U* tests, *p* = .264), and between *Pseudomonas*‐infected aphids and uninfected aphids (Mann–Whitney‐*U* tests, *p* = .650). However, the densities of *Acinetobacter* (*r* = .1624, *p* = .0870), *Microbacterium* (*r* = −.1589, *p* = .1038), and *Pseudomonas* (*r* = −.3273, *p* = .0589) were not found with any impact on the density of *Buchnera* bacterium (Appendix S4).

Coinfection with facultative symbionts was common in *A*. *gossypii*. It was found that 10.44% and 1.37% aphids were recorded with two or three facultative symbionts simultaneously. Only one aphid was coinfected with four facultative symbionts. The maximum coinfection type was of *Arsenophonus* and *Hamiltonella*, accounted for 4.44%, followed by coinfection of *Arsenophonus* and *Microbacterium* (2.15%) and coinfection of *Arsenophonus* and *Acinetobacter* (1.44%).

**FIGURE 4:**
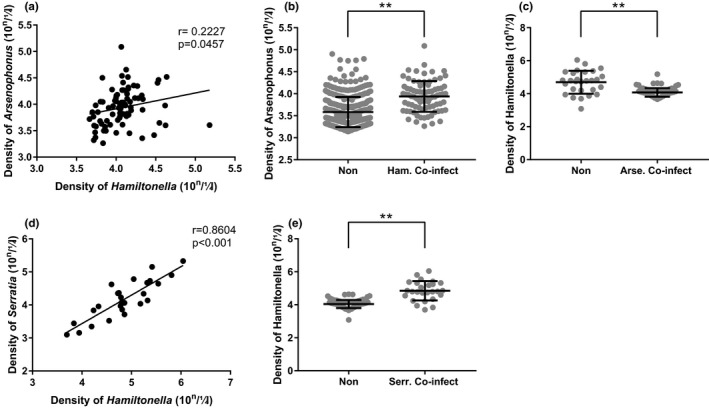
The relationship between facultative symbionts. The correlation between *Arsenophonus* and *Hamiltonella* copy numbers (a), *Arsenophonus* copy numbers in aphid co‐infection with *Hamiltonella* (Ham.Co‐infect) and aphids no co‐infection with *Hamiltonella* (Non) (b), *Hamiltonella* copy numbers in aphid co‐infection with *Arsenophonus* (Arse.Co‐infect) and aphids no co‐infection with *Arsenophonus* (Non) (c). The correlation between *Serratia* and *Hamiltonella* copy numbers (d), *Hamiltonella* copy numbers in aphid co‐infection with *Serratia* (Serr.Co‐infect) and aphids no co‐infection with (Non) (e). Scatter dot plot with mean ± standard deviation. **p < .01, data were analysis by Mann–Whitney *U* test

The *Hamiltonella*‐infected aphids have recorded with higher coinfection frequency with other facultative symbionts (96.33%), followed by *Pseudomonas*‐infected aphids (55.38%). The density of *Hamiltonella* detected with obvious positive correlation with *Arsenophonus* density (*r* = .2227, *p* = .0457) and *Serratia* (*r* = .8604, *p* < .001) in *A*. *gossypii* (Figure 4a,d). The *Arsenophonus‐*infected aphids have recorded with a higher *Arsenophonus* titer level when observed with *Hamiltonella* (Mann–Whitney‐*U* tests, *p* < .001), and *Hamiltonella‐*infected aphids have lower *Hamiltonella* titer level when detected with *Hamiltonella* (Mann–Whitney‐*U* tests, *p* < .001) but has a higher *Hamiltonella* titer level when noticed with *Serratia* (Figure 4b–e). The aphids observed with *Hamiltonella* were all coinfected with *Arsenophonus* in Xinjiang population except one aphid, 50% aphids, collected from North China, coinfected with *Arsenophonus,* whereas coinfection was not recorded in Hainan‐collected aphid samples.

### 3.3 Assessment of infection frequency in population of aphids of Henan, Hainan and Xinjiang

#### 3.3.1 Aphid samples collected from cotton plant

Multivariate analysis showed that there were significant differences in bacterial community compositions among geographic populations feeding on cotton (ADONIS: *F*_2,433_ = 37.587, *R* ^2^ = .148, *p* = .001; ANOSIM: *R* = .022, *p* = .001). There were significant differences in infection frequency of *Hamiltonella* and *Arsenophonus* belonging to the three geographic populations collected from cotton plants (χ^2^ = 91.909, *p* < .001 and χ^2^ = 72.767, *p* < .001, respectively). The *Hamiltonella* infection frequency was higher in Xinjiang (36.96%, *n* = 184) and Hainan (22.86%, *n* = 35), although very low infection frequency was recorded in Henan (0.46%, *n* = 217). *Arsenophonus* infection frequency was higher in Xinjiang (75.54%) and Henan (46.08%), whereas lower infection frequency was observed in Hainan (5.71%). *Microbacterium* infection frequency was higher in Henan (29.95%). Hainan populations have higher *Serratia* and *Pseudomonas* infection frequencies among these three geographic populations, 31.43% and 34.29%, respectively, whereas in other two geographic populations, the infection frequencies were less than 6.60%, even have not recorded any infection of some symbiotic bacterial species in some aphid populations.

**FIGURE 5:**
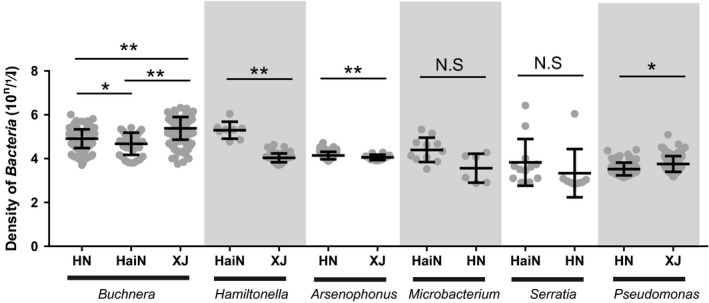
The densities of symbiotic bacteria in different *Aphis gossypii* geographic populations collected on cotton. The symbiotic bacteria copy numbers in Henan province (HN), Hainan province (HaiN), and Xinjiang province (XJ) were showed, scatter dot plot with mean ± standard deviation. *p < .05, **p < .01, N.S = no significant difference, data were analysis by Mann–Whitney *U* test

The abundance of *Buchnera* and four facultative symbionts were significantly dissimilar in different geographical aphid populations. *Buchnera* has highest abundance in Xinjiang populations, which was 1.70‐fold and 5.04‐fold more than Henan (Mann–Whitney‐*U* tests, *p* < .001) and Hainan populations (Mann–Whitney‐*U* tests, *p* < .001), respectively. *Hamiltonella* has a higher titer level in the Hainan population (*n* = 8), which was 23.84‐fold greater than in Xinjiang (*n* = 68). The titer level of *Serratia* and *Pseudomonas* was maximum in Hainan (*n* = 11 and *n* = 12, respectively) than that in Henan populations (*n* = 6 and *n* = 8, respectively), which were 6.59‐fold and 1.79‐fold larger, respectively. The abundance of *Arsenophonus* was 1.96‐fold greater in the Xinjiang population than that in Henan (Mann–Whitney‐*U* tests, *p* < .001) (Figure 5).

#### 3.3.2 Aphid samples collected from hibiscus plants

Hibiscus is the main primary host of cotton aphid. During the occurrence, cotton aphid was collected from 24 geographic populations from hibiscus plants grown in North China. Samples from all the geographic populations contained infected aphids with *Buchnera* and *Arsenophonus*. Fifteen of 24 samples collected from various geographic populations contained aphids infected with *Microbacterium*. The other four facultative symbionts detected ranged from 33.33% to 45.83% in 24 geographic studied populations (Appendix S5). Multivariate analysis showed that there were significant differences in bacterial community compositions among geographic populations feeding on hibiscus (ADONIS: *F*_23,1,073_ = 7.675, *R* ^2^ = .141, *p* = .001; ANOSIM: *R* = .083, *p* = .001).

Generally, the aphid's infection frequency with facultative symbionts were at a low level in each geographic population. Average aphids (18.97%) were recorded with *Arsenophonus*, with highest infection frequency (44.90%), although lowest infection frequency remained at 4.88%. About 4.87% aphids were infected with *Microbacterium*, 6.68% were observed with *Serratia* and 2.97% aphids recorded with *Pseudomonas*. In *Hamiltonella*‐infected 10 populations, 1.52%–13.98% aphids were infected with *Hamiltonella*. All the 7 populations collected in Henan Province were infected with *Acinetobacter*, and 3 10 populations collected in Shandong Province were infected with *Acinetobacter*, but the infection frequency in each population was lower than that in the Henan population (Appendix S5).

**FIGURE 6:**
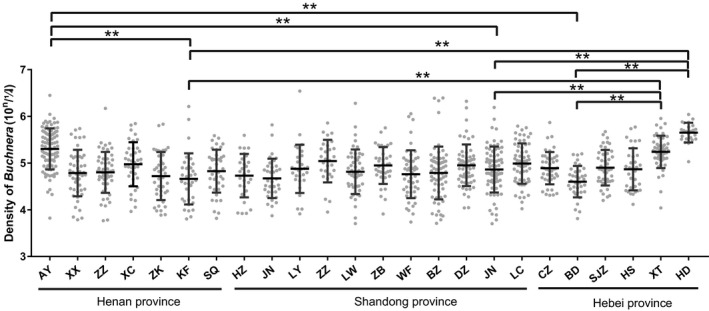
The densities of *Buchnera* in different *Aphis gossypii* geographic populations collected on hibiscus. The *Buchnera* copy numbers in Anyang city (AY), Xinxiang city (XX), Zhengzhou city (ZZ), Xuchang city (XC), Zhoukou city (ZK), Kaifeng city (KF), Shangqiu city (SQ), Heze city (HZ), Jining city (JN), Linyi city (LY), Zaozhuang city (ZZH), Laiwu city (LW), Zibo city (ZB), Weifang city (WF), Binzhou city (BZ), Dezhou city (DZ), Jinan city (JN), Liaocheng city (LC), Cangzhou city (CZ), Baoding city (BD), Shijiazhuang city (SJZ), Hengshui city (HS), Xingtai city (XT), and Handan city (HD) were showed, scatter dot plot with mean±standard deviation. **p < .01, others showed no significant difference, data were analysis by Mann–Whitney *U* test

The aphids collected from Handan city (Hebei Province) had a higher abundance of *Buchnera*, 9.47‐fold higher than Baoding city population, whereas the Baoding city population has the lowest abundance of *Buchnera* among 24 geographic populations. Interestingly, three populations recorded with maximum *Buchnera* copies/abundance were from adjacent regions, Handan city, Xingtai city (Hebei Province), and Anyang city (Henan Province), with a significantly higher *Buchnera* abundance than Baoding city, Hebei Province (Mann–Whitney‐*U* tests, *p* < .001); Jining city (Mann–Whitney‐*U* tests, *p* < .001); and Kaifeng city, Henan Province (Mann–Whitney‐*U* tests, *p* < .001), and no significant difference with other cities (Figure 6).

### 3.4 Assessment of infection frequency within geographic populations of aphids collected during different time periods

#### 3.4.1 Three‐year infection comparison among geographic populations from Anyang, Henan

**FIGURE 7:**
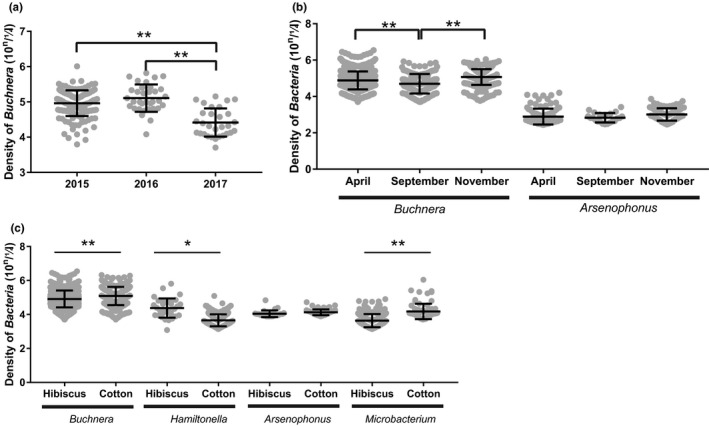
The densities of symbiotic bacteria in *Aphis gossypii* collected at different times and on different hosts. The copy numbers of *Buchnera* in *A. gossypii* from Anyang city during 2015–2017 (a), the copy numbers of *Buchnera* and Acinetobacter in *A. gossypii* collected on hibiscus in April September November (b), the copy numbers of *Buchnera*, *Hamiltonella*, *Arsenophonus*, and *Microbacterium* in *A. gossypii* collected on hibiscus and cotton (c) were showed, scatter dot plot with mean ± standard deviation. *p < .05, **p < .01, others showed no significant difference, data were analysis by Mann–Whitney *U* test

The infection frequency of facultative symbionts in cotton aphids has significant difference from 2015 to 2017 from Anyang, Henan (ADONIS: *F*_2,214_ = 21.197, *R* ^2^ = .165, *p* = .001; ANOSIM: *R* = .226, *p* = .001). About 50% aphids were found infected with *Arsenophonus* both in 2015 and 2016, but only 13.33% individuals in 2017 (Fisher's exact test, both *p* < .001). There were 41.83% aphids infected with *Microbacterium* in 2015, but almost none of infected‐aphid was detected in 2016 and 2017 (Appendix S5). The abundance of *Buchnera* has no significant difference between 2015 and 2016 (Mann–Whitney‐*U* tests, *p* = .071), although 3.13‐fold (Mann–Whitney‐*U* tests, *p* < .001) and 4.62‐fold (Mann–Whitney‐*U* tests, *p* < .001) higher than in 2017, respectively (Figure 7a).

#### 3.4.2 Infection comparison among geographic populations from North China

*Aphis gossypii* were collected from hibiscus plants in three varying time periods in 2016. Multivariate analysis showed that there were significant differences in bacterial community compositions among populations feeding on hibiscus at three periods (ADONIS: *F*_2,1094_ = 24.490, *R* ^2^ = .043, *p* = .001; ANOSIM: *R* = .062, *p* = .001). About 7 bacteria were detected in sampled aphids, except *Hamiltonella* in September and *Acinetobacter* in November. There were about 15% aphids infected with *Arsenophonus* in April and September, lower than the infection frequency in November, 30.82% (χ^2^ = 11.844, *p* = .001 and χ^2^ = 29.124, *p* < .001, respectively). There were 16.00% aphids infected with *Acinetobacter* in April, infection frequency dropped up to 1.41% in September, and no infected aphid was detected in November. The highest infection frequency with *Microbacterium* was detected in November, 6.56%; meanwhile, 1.08% and 0.70% aphids were found infected with *Microbacterium* in April and September, respectively. *Serratia* has highest infection frequency in September, 6.34%, and 1.54% and 3.93% individuals were infected with *Microbacterium* in April and November, respectively.

Both *Buchnera* and *Arsenophonus* abundance were lowest in September. The *Buchnera* abundance in April and November was 1.56‐fold (Mann–Whitney‐*U* tests, *p* < .001) and 1.76‐fold (Mann–Whitney‐*U* tests, *p* < .001) higher than that in September, respectively. The *Arsenophonus* abundance in April and November was 1.94‐fold (Mann–Whitney‐*U* tests, *p* = .811) and 1.71‐fold (Mann–Whitney‐*U* tests, *p* = .030) higher than that in September, respectively (Figure 7b).

### 3.5 Assessment of infection frequency among aphid populations collected from various host plants

All bacteria were detected in *A*. *gossypii* feeding on cotton and hibiscus. Multivariate analysis showed that there were significant differences in bacterial community compositions between aphids feeding on cotton and hibiscus (ADONIS: *F*_1,1531_ = 33.916, *R* ^2^ = .022, *p* = .001; ANOSIM: *R* = .062, *p* = .001). The frequency of four bacterial infection was higher in aphids feeding on cotton than those feeding on hibiscus. These infection frequencies were *Hamiltonella* (17.66% in aphid on cotton vs. 2.92% in aphids on hibiscus, χ^2^ = 102.683, *p* < .001), *Arsenophonus* (55.28% vs. 19.87%, χ^2^ = 186.421, *p* <.001), *Microbacterium* (17.89% vs. 2.55%, χ^2^ = 114.740, *p* < .001), and *Pseudomonas* (4.59% vs. 1.28%, χ^2^ = 15.771, *p* < .001). The infection frequency of *Acinetobacter* was higher in aphids feeding on hibiscus than aphids feeding on cotton (9.66% vs. 1.38%, χ^2^ = 31.635, *p* < .001), and *Serratia* infection frequency has no significant difference between aphids feeding on hibiscus and cotton (2.83% vs. 4.36%, χ^2 ^= 2.321, *p* = .128).

The abundance of *Buchnera* in aphid populations feeding on cotton was 1.48‐fold higher than aphid populations feeding on hibiscus (Mann–Whitney‐*U* tests, *p* < .001). *Microbacterium* abundance was 1.12‐fold higher in the population feeding on cotton than aphid populations feeding on hibiscus (Mann–Whitney‐*U* tests, *p* = .002). Aphids feeding on hibiscus have 1.47‐fold higher *Hamiltonella* abundance than aphids feeding on cotton (Mann–Whitney‐*U* tests, *p* = .029). There was no significant difference between *Arsenophonus* abundance between aphids feeding on hibiscus and aphids feeding on cotton (Mann–Whitney‐*U* tests, *p* = .277) (Figure 7c).

## 4 DISCUSSION

Symbiotic bacteria are necessary for the survival, reproduction, host adaption, and resistance to biotic or abiotic stresses of most insects, especially for the sap‐sucking insect‐like aphids (Hosokawa et al., 2010; Ley et al., 2008; Moran, 2007; Teixeira et al., 2008). First, we used the 16S rRNA gene sequencing on Illumina platforms to compare the differences of cotton aphid symbiotic bacteria between Xinjiang and Henan populations and identify the dominant symbiotic bacteria. There is no doubt that the primary symbiont *Buchnera* exists in the cotton aphids in a dominant proportion. In this study, the relative abundance of *Buchnera* was more than 74% in the aphid population from both locations Henan and Xinjiang; however, the linear distance between the two places exceeds 2,500 km. Aphids from Henan Province have a higher proportion of facultative symbionts than aphids collected from Xinjiang Province, and both richness estimates and biodiversity of bacteria were higher in Xinjiang. PCA studies showed some aphid population in Xinjiang has large variations. Henan is warm temperate‐subtropical area, whereas Xinjiang has wide range of temperature differences between days and nights. Varying environmental conditions may be the cause of these differences during the spread of cotton aphid populations (Dunbar et al., 2007).

Symbiont abundance were previously measured in whole insects as the number of symbiotic bacteria per aphid genome using qPCR and normalized by single‐copy gene abundance (Chong & Moran, 2016). In this study, adult aphids were used, during the early stage of adult aphid, and the number of symbiotic bacteria was basically stable (Ayoubi et al., 2020), so the age of adult aphids has little effect on the community of symbiotic bacteria. When aphids reproduce via viviparous parthenogenesis, ovaries occupy a large proportion of the female body, such as the last stage maturing embryo length was longer than 0.8 mm compare with the mother's body size 4.0–5.0 mm of *Acyrthosiphon pisum* (Rabatel et al., 2013). During development, the embryo of *A*. *pisum* just receives a small proportion of *Buchnera* from the mother at the beginning, immediately following rapid multiplication of the bacterium (Wilkinson et al., 2003). This causes the uneven distribution of symbiotic bacteria among the adult aphids, so we used the abundance of symbiotic bacteria in a single aphid to measure its richness. The DNA of each adult aphid for qPCR analysis was extracted according to a consistent method to ensure that the same DNA yields for each individual. To ensure the consistency of extraction, samples with lower concentrations were discarded. Aphid samples having DNA concentrations near 30–40 ng/μl were used for qPCR studies.

So far, the infection of symbiotic bacteria in the natural population of cotton aphids is still poorly understood. In this study, the abundance of 7 bacteria in individual aphids were appraised by using qPCR. The seven bacteria were *Buchnera*, *Arsenophonus*, *Acinetobacter*, *Hamiltonella*, *Serratia*, *Microbacterium*, and *Pseudomonas*. *Arsenophonus*, *Acinetobacter*, *Hamiltonella*, and *Serratia* were the top genera, recorded in aphids, collected from Xinjiang and Henan. Comparable studies also show a similar trend in relative abundance of the bacteria in cotton aphid (Ayoubi et al., 2020; Tian et al., 2019; Xu et al., 2020; Zhang, Pan, et al., 2016; Zhao et al., 2016). The abundance of the infrequent bacterium *Pseudomonas* was higher in some cotton aphids (Gallo‐Franco et al., 2019). *Pseudomonas* is considered as a widespread aphid pathogen, and *A. pisum* can reduce the infection by avoiding the highly virulent strain of *Pseudomonas* (Hendry et al., 2018). *Microbacterium* belongs to Actinobacteria, which is widely present in air, soil, water, and plants, as well exists in guts of larvae and adult insects (Dantur et al., 2015), and has not influenced by spirotetramat insecticide used against cotton aphids (Zhang, Pan, et al., 2016). In the current study, there was a group of symbiotic bacteria, which accounts for a large proportion in cotton aphids, identified to family level, *Enterobacteriaceae*, and excluded due to ineffective detection by specific primers, utilized against *Exiguobacterium*.

The infection frequency of symbiotic bacteria in cotton aphids draws different conclusions in different research methods. Through a wide range of single‐individual qPCR studies, the presence of the main symbiotic bacteria of *A*. *gossypii* was basically outlined. Using the diagnostic PCR method, about 44.58% (*n* = 1,200) aphids were infected with *Arsenophonus* in cotton aphids from the Nanjing population (Tian et al., 2019), which is significantly higher than infection frequency in this study (29.94%, *n* = 1533) (χ^2^ = 62.356, *p* < .001). High‐throughput 16S rRNA sequencing showed 5.45% (*n* = 110) *A*. *gossypii* were infected with *Hamiltonella*, and most of them were collected from Beijing, North China, and Xinjiang (Xu et al., 2020). The *Hamiltonella* infection frequency was not significant in our study (χ^2^ = 0.432, *p* = .511), indicating that *Hamiltonella* infection frequency in the natural population of cotton aphids was low. Other studies showed that North American *A*. *pisum* populations have higher infection frequencies of *Hamiltonella* (32.39%, *n* = 318); it may reflect those aphid populations have different needs, depending on the benefits provided by *Hamiltonella*, which needs a balance with fitness costs of the bacterium (Hafer‐Hahmann & Vorburger, 2020; Vorburger & Gouskov, 2011). More than 36% *Serratia* infection frequency has reported in pea aphids (Parker et al., 2017), but in current studies related to the natural population of cotton aphids, *Serratia* frequency was low, significantly lower than previous study (χ^2^ = 144.405, *p* < .001) (Xu et al., 2020). Different studies showed different infection frequencies of *Serratia* and *Arsenophonus* in cotton aphids but not *Hamiltonella*; the reason for this was the infected aphids have low abundance of *Arsenophonus* and *Serratia*, existing among the aphid samples. In this study, DNA was first diluted to 10‐fold for qPCR. Aphids having bacterial abundance lower than the lowest point of the standard curve were considered not infected with this bacterial, resulting in a relatively lower infection frequency than diagnostic PCR direct use original DNA solution. Although *Hamiltonella‐*infected individuals always have a high abundance in natural cotton aphid populations, few aphids were considered undetected individuals in data processing.

Based on resources, survival niches, and interaction between aphids and bacterial symbionts, there are complex relationships among them, known as metabolic tug of war (Smith & Moran, 2020). Not all facultative symbionts can affect the abundance of *Buchnera* in cotton aphids. Subsequently, in aphids infected with *Hamiltonella*, the abundance of primary *Buchnera* is increased, but in infected aphids, there was a negative correlation of *Hamiltonella* on the abundance of *Buchnera* in cotton aphids (Xu et al., 2020; Zhang, Luo, Wang, et al., 2019). The infection of *Arsenophonus* in cotton aphids showed different results, for example, when infected with *Arsenophonus*, the abundance of *Buchnera* reported to be increased (Tian et al., 2019), and continuously increases with the increase in *Arsenophonus* abundance. The opposite coinfection relationship was showed by using high‐throughput 16S rRNA sequencing (Xu et al., 2020) as the use of relative abundance was easy in incorrectness due to the inconsistency of the total abundance. In several aphid species, loss of genes in *Buchnera* complements in the *Serratia* genome, and *Buchnera* and *Serratia* reported to be evolved together during the biosynthesis of tryptophan (Lamelas et al., 2011; Monnin et al., 2020). *Serratia* has a low infection level in the natural cotton populations, indicating that it is not necessary for the survival of cotton aphids. But *Serratia* infection can influence the *Buchnera* densities in cotton aphids; this implies that there may be a close interaction between them.

The low infection frequency may indicate that cotton aphids were more likely to acquire symbiont bacteria through horizontal transfer because of more complicated coinfection of secondary bacterial symbionts. Nearly 100% coinfection of *Hamiltonella* and *Arsenophonus* was recorded in Xinjiang population, where cotton aphids have to deal with more complex biological and abiotic factors. A previous study showed that *Arsenophonus* together with *Hamiltonella* contributed to the fitness of *A*. *gossypii* by enhancing its performance (Ayoubi et al., 2020). Recent research shows *Hamiltonella* also can have an effect on insecticide resistance of aphids (Li et al., 2020) or can alter the dynamics of host metabolic interactions with co‐occurring microorganisms (Blow et al., 2020). Costs and benefits of coinfection have influenced within‐host interactions between these symbionts, resulting in coinfection dynamic changes within natural populations (Russell et al., 2013).

Geographical distribution affects the common bacterial symbiont in insects (De Cock et al., 2020; Pan et al., 2012). Cotton is widely grown in China and serves as the main secondary host of cotton aphids. Hibiscus is the main primary host. *A*. *gossypii* were collected from cotton from three regions with obvious climate differences. In the studied regions, cotton aphids have a holocyclic life cycle, that is, in Henan and Xinjiang and reproduce continuously by apomictic parthenogenesis in Hainan, recorded with significant difference in bacterial symbiont infection frequencies and abundance among the three geographic populations of aphids. This situation also arises in hibiscus populations collected at different times and regions. *Hamiltonella* can confer tolerance to high temperatures in *A*. *pisum* (Russell & Moran, 2006), and some *Serratia* are known to be involved in the detoxification of insecticides (van den Bosch & Welte, 2017), or it could increase insect susceptibility to exposed insecticides (Skaljac et al., 2018). Although *A*. *gossypii* were collected from hibiscus, infection frequencies and abundance remained greatly different among various geographic populations. The symbiotic bacteria may help the host organism to improve adaptability, affected by environmental and/or historical factors (Tsuchida et al., 2002).

In this study, *A*. *gossypii* were collected from hibiscus in April, September, and November from North China, where eggs of the aphid hatched on the host plant—hibiscus—and later, after the month of April, migrated to the host plant in summer. There were some cotton aphids still hosting hibiscus plants in September, and alate adults return to the hibiscus to mate and oviposit in November (Xia, 1997). The natural population of cotton aphids was suppressed to a small number several times a year because of the natural environment, especially due to rain and or absence of food plants (Hu et al., 2017; Liu et al., 2017), so migration of aphids among host plants occurs several times a year. There were differences in infection frequencies of bacterial symbionts among geographic populations, and most of the facultative symbionts spread more likely through horizontal transfer and can pass through plants (Henry et al., 2013), indicating that *A*. *gossypii* migrate in a small area. The infection of facultative symbionts, in the aphids, remained significantly different among different years from the same place with significant fluctuations in abundance. It shows that there is no obvious dominant population in the aphid population, in response to environmental changes; populations and symbiotic bacteria have great variability.

In conclusion, using the 16S rRNA gene sequencing, the dominant symbiotic bacteria were identified, and the abundance of symbiotic bacteria was estimated using qPCR in natural populations of *A. gossypii*. The low infection frequency of facultative symbionts in the natural population of cotton aphids were found across host plants, geographic area, and time periods. The infection of *Serratia*, *Hamiltonella*, and *Arsenophonus* can affect the density of *Buchnera* with different modes, and there was also obvious interaction between facultative symbionts. By analysis of the symbiotic bacteria from different geographic areas, we speculate that *A*. *gossypii* migrate in a small area.

## CONFLICT OF INTEREST

The authors declare that they have no conflict of interests.

## AUTHOR CONTRIBUTIONS

**Shuai Zhang:** Conceptualization (equal); Data curation (equal); Funding acquisition (equal); Methodology (equal); Writing‐original draft (equal); Writing‐review & editing (equal). **Honghua Su:** Writing‐review & editing (equal). **Weili Jiang:** Data curation (equal). **Daowu Hu:** Resources (equal). **Intazar Ali:** Writing‐review & editing (equal). **Tianxing Jing:** Methodology (equal). **Yizhong Yang:** Supervision (equal); Writing‐review & editing (equal). **Xiaoyan Ma:** Project administration (equal); Resources (equal); Writing‐review & editing (equal).

## ETHICS APPROVAL

This study does not contain any studies with human participants or vertebrate performed by any of the authors. Cotton aphids are invertebrate insects and, according to the IUCN criteria, are not considered as endangered or protected species. Cotton aphid samples were collected from farmer's fields and ornamental hibiscus after obtaining their permission.

## INFORMED CONSENT

Informed consent was obtained from all individual participants included in the study.

## Supporting information

Appendix S1

Appendix S2

Appendix S3

Appendix S4

Appendix S5

## Data Availability

All raw sequences were deposited in the NCBI Sequence Read Archive under accession number SRA Accession no. PRJNA725955.
