# Supplementary material for: Symbiotic microbial studies in diverse populations of Aphis gossypii, existing on altered host plants in different localities during different times
Source: Ecol Evol. 2021 Sep 23;11(20):13948–60. doi: 10.1002/ece3.8100 (PMC8525075; doi:10.1002/ece3.8100)
Supplement: Supplementary file 1 — Appendix S1 [file ECE3-11-13948-s004.docx]

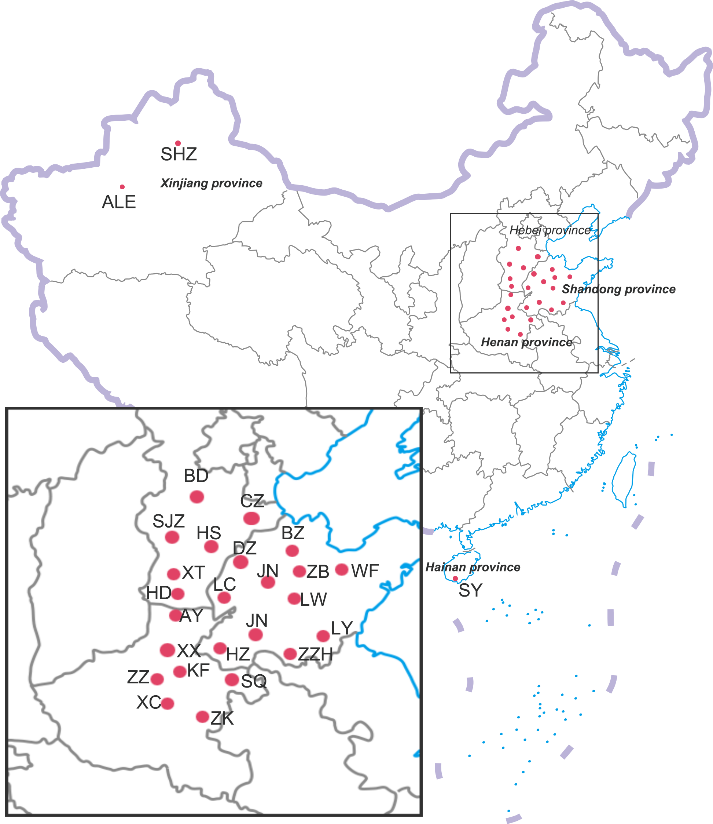


Anyang city (AY), Xinxiang city (XX), Zhenzhou city (ZZ), Xuchang city (XC), Zhoukou city (ZK), Kaifeng city (KF), Shangqiu city (SQ), Heze city (HZ), Jining city (JN), Linyi city (LY), Zaozhuang city (ZZH), Laiwu city (LW), Zibo city (ZB), Weifang city (WF), Binzhou city (BZ), Dezhou city (DZ), Jinan city (JN), Liaocheng city (LC), Cangzhou city (CZ), Baoding city (BD), Shijiazhuang city (SJZ), Hengshui city (HS), Xingtai city (XT), Handan city (HD), Sihezhi city (SHZ), Alaer city (AIE), Sanya city (SY).
